# Experimental Evaluation of Interfacial Surface Cracks in Friction Welded Dissimilar Metals through Image Segmentation Technique (IST)

**DOI:** 10.3390/ma11122460

**Published:** 2018-12-04

**Authors:** Gulam Mohammed Sayeed Ahmed, Ali Algahtani, Essam R. I. Mahmoud, Irfan Anjum Badruddin

**Affiliations:** 1 Department of Mechanical Engineering, College of Engineering, King Khalid University, PO Box 9004, Abha 61413, Asir, Kingdom Saudi Arabia; alialgahtani@kku.edu.sa (A.A.); irfan@kku.edu.sa (I.A.B.); 2 Research Center for Advanced Materials Science (RCAMS), King Khalid University, PO Box 9004, Abha 61413, Asir, Kingdom Saudi Arabia; 3 Department of Mechanical Engineering, Faculty of Engineering, Islamic University of Madinah, Medina 42351, Saudi Arabia; emahoud@iu.edu.sa; 4 Central Metallurgical Research and Development Institute (CMRDI), 12422 Cairo, Egypt

**Keywords:** friction welding, Al-Cu welded joint, dissimilar joint, image segmentation technique, response surface methodology

## Abstract

Surface cracks on the friction welded interface of dissimilar metals are one of the earliest indications of degradation of the joint, which is a critical aspect for the welding strength. By manual inspection of the friction welded joint, observations of irregularities, porosity voids, crack lengths, cracked surfaces, and depth penetrations of two dissimilar metals can be made. Manual inspection purely depends on a quality expert’s experience of quantitative analysis and knowledge. In this research, an attempt has been made to effectively utilize the image segmentation technique (IST) in the estimation of the welded surface quality of a dissimilar joint by friction welding. The bonding strength between dissimilar metals in friction welding is more dependent on the coefficient of friction between the metallic surfaces. To demonstrate the capability of the image segmentation technique, experiments were conducted with various parameters, such as friction pressure, friction time, coefficient of friction, and torque speed of the rotating work piece. The effect of the coefficient of friction on friction welded surface quality by considering process parameters is estimated by using the proposed technique. Experiments were validated and the results claimed that the proposed image processing approach is efficient in fractured surface crack detection, reducing the computation cost, and providing a high-speed method with greater accuracy in the identification of welded surface defects. It was found that the friction coefficient is dependent mostly on the friction pressure and friction time. Its values range from 0.21 to 0.71, with the highest value of friction pressure at 120 MPa and 500 rpm. The present work deals with the detection of welding defects by means of segmentation based analysis of the welded interface. This method has a significant improvement in the fractured surface, crack detection, and non-welded areas’ detection in terms of pixels at the desired region, and is easy when compared to conventional detection techniques by using an operator’s decisions.

## 1. Introduction

In many industrial applications, friction welding (FW) processes can replace traditional joining methods. This is due to its capability of high performance in joining different ferrous and non-ferrous metals [1]. One of the important aspects in this process is that it is considered as a solid-state process. The maximum temperature attained during welding does not exceed the melting points of one of the two metals. Friction welding of dissimilar metals utilizes heat generated due to friction between the one rotating metallic surface and another held stationary in a chuck [2]. Continuous friction on the stationary metallic surface is maintained so that the heat generated will continue to rise to a critical temperature below the melting point and both metals are welded. The friction welding process has attracted many researchers due to its solid-state process and short welding time, which can reduce the thermal compatibility between the base parent materials [3]. Sound quality joints were thus proved to be stronger from friction welding of dissimilar joints [4,5,6,7,8,9]. The mechanical properties of the welded joints can be improved by reducing the interfacial metallic thickness of the welded zone of dissimilar metals. The thickness of the interfacial metallic layer can be minimized by optimizing the process parameters and metallic composition of the weld metal [10]. Paventhan et al. explored the optimization and predicted the process parameters that affect the aluminum-steel dissimilar joint strength and quality [11,12,13,14,15]. The most critical aspects in friction welding when compared with other welding processes are the high joint strength and lesser processing time. Many researchers [16,17,18] have compared dissimilar joints obtained by friction welding and the friction stir welding process [19,20]. The effect of process parameters, such as friction pressure, on the properties of hot rolled super alloys has been reported by Hakan Ates et al. [21]. Afes Hakan and Sathiya Paulraj et al. [22,23] explained that the grains of the friction welded area are refined at the welded interface due to the severe plastic deformation, which results in enhancement of mechanical properties [22,23]. Gontarz et al. [24] explored the effect of process parameters in joining Al-Mg at elevated temperatures and estimated the coefficient of friction. In this research work, the image segmentation technique was used to quantify the flaws at the welded interface and establish a correlation within the two dissimilar metals to be welded to predict the optimal operating process parameters, which were experimentally validated. Then, tests were carried out to check the weld quality in terms of crack detection and the fractured surface of the welded joint. It was demonstrated that a good weld quality joint can be obtained by using optimized process parameters. The proposed methodology of image segmentation was successfully implemented in evaluating the fractured surface and crack detection on the welded surface. The experimental results showed that the effect of the coefficient of friction plays a vital role in the welding quality. In the present work, traditional solid state welding, i.e., the friction welding process, was applied.

## 2. Experimental Work

**Figure 1:**
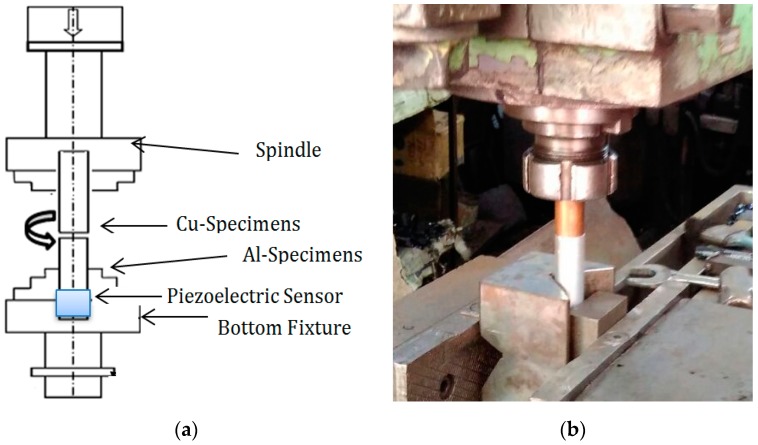
(**a**) Schematic layout of the friction welding process. (**b**) Experimental set-up.

This present research work investigates the influence of the friction welding parameters, pressure, rotational speed, and friction contact time, on friction welding of aluminum (Al), brass, and copper (Cu) on the coefficient of friction at the interface, which directly affects the quality of welded joints. In Figure 1, the schematic layout and experimental set up of the friction welding process is shown, consisting of joining Al-6065, placing it in a fixture, and the rotated counterpart is copper. The initial phase of the friction welding process is termed as the friction time period. A fixture brings both the specimens together till the metal surfaces comes in contact and the friction between the rotating and fixed metallic surface tends to start and generate a large amount of heat, which reaches a high temperature that is close to, but not exceeding, their melting points. In the consecutive phases, termed as the upsetting period, friction pressure will be increased for a particular time. In this stage, the welded joint was created. The chemical composition and the mechanical properties of Al-Cu metals used in the FW process are presented in Table 1 and Table 2.

The metal specimens used in the friction welding experiments were cylindrical rods of a 100 mm length and 12 mm diameter. The cylindrical metal specimens were maintained at the exact dimensions to a 12 mm diameter by the turning process. The surfaces of the specimens to be welded were also cleaned with acetone prior to welding. Experiments were conducted using a machine adjusted with a range of nine variable rotational speeds of 250, 375, 500, 750, 1000, 1250, 1350, 1400, and 1500 rpm. Friction torque and friction force were recorded with piezoelectric type sensors located at a fixed fixture in which the stationary specimens were allocated. Welded zone temperatures were recorded with a FLUKE company (Everett, WA, USA) product thermal sensor during different process parameters of the FW process. The high sensitivity of the sensor ensured correct readings with a measurement accuracy of 20 °C. The friction welding machine used operated with controlled accuracy and good repeatability of the friction welding parameters. The spindle speed was maintained by an alternating current, and friction forces were read by a piezeo-electric sensor. The spindle motor capacity was 30 W with 3 Phase AC and its operating speed can be varied from 1 to 1000 rpm. All the experimental data with a possible combination of welding parameters were recorded. The machine had a stroke length of 350 mm and a maximum friction force of 300 kN was applied. The spindle speeds were varied in steps up to 500 rpm. Nine different combinations of friction welding parameters were performed as given in Table 3. The friction welding process was carried out at pressure forces of three values of 55, 85, and 120 MPa. The surface quality and integrity of the welded specimens were examined by the IST technique.

## 3. Image Segmentation Technique

**Figure 2:**
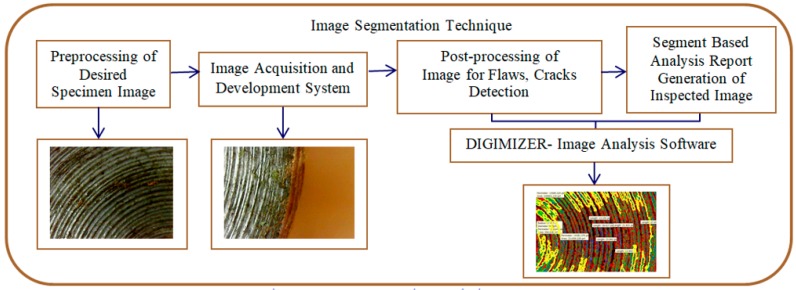
Procedural steps of the image segmentation technique.

There are certain industrial requirements that define their quality standards to meet exactly the customer’s requirements and specifications. It strongly needs specific inspection and testing to be performed on final products in mass or batch productions. The components are inspected by conventional or non-destructive techniques. The scenario image segmentation technique is considered to be a powerful tool for accurate data interpretation in assuring the confirmed process parameters for quality-based products. Figure 2 illustrates the procedural steps of the image segmentation technique. Many of the latest inspection systems are based on processing an image taken from an inspected product.

Image analysis plays an important role in evaluating the desired process by the image acquisition system. In the present research work, image segmentation techniques were applied to enhance and analyze the resultant image and with the help of a knowledge based database in deciding whether the product could pass the quality inspection tests. The acquired images were captured and transferred to the computer for further processing by using the DIGIMIZER Image Analysis Software (Version 5.3.4, Med-Calc, Ostend, Belgium) for automatic inspection. The acquired images were pre-processed to be enhanced and possible flaws as segments were evaluated and analyzed. The main objective of image preprocessing is to improve the visibility of the captured images to a suitable scale for the human eye. The segmentation process is one of the important image processing techniques for an inspection system. It is the process of dividing and clustering the images into areas of desired segment analysis. A segmentation based routine or algorithm for a friction welding image needs to be bifurcated, like a porous region, edging, surface cracks, crack length, peaks, valleys, and gas inclusions, etc. Particular threshold values are designated for segments in terms of pixels accumulated in the desired region. The quantification of images for crack detection are expressed in terms of the pixels. Once the image segmentation process is completed, the resultant images in terms of segments are analyzed. This is a classifying process of different defects and it is considered a feature or pattern recognition. A general feature extraction and recognition system consists of a segment processor, feature detection unit, and classification unit. From the input file flaws, the pattern and segmented objects were traced. The feature detection unit extracts data information in terms of pixels. The classification unit categorizes features and patterns. In the present work, analysis of the segmented image can be regarded as surface crack detection. The segmentation process was characterized by three different grey levels. The first segmentation level feature was extracted from the desired region. This feature extraction is considered as a surface defect; surface crack length. Subsequently, features were extracted and the repeated procedure covered the desired region of inspection. The DIGIMIZER surface analysis software starts from a fixed location as the starting point in terms of pixels of the current feature to extract. Then, all similar featured pixels close to the starting pixel were evaluated, if a pixel was found to be of the same grey-level as the first feature, then it was confirmed to be of the same featured category and the pixels were evaluated for the total region. Conventional methods were applied for the quantification of similar features, such as porosity area, crack surfaces, crack length, perimeter, feature brightness, etc. the brightness will be expressed in terms of green, blue, and red colors. Once the pattern or features were traced, they were analyzed and compared with previously known patterns for a final decision.

## 4. Mathematical Modeling of Input Heat Energy, $H_{f}$, and Coefficient of Friction, $\mu_{f}$

The obtained time-relationships of pressure force, friction torque, and temperature were used for determining the value of the friction coefficient at a given temperature. The friction coefficient was calculated from the formula:

(1) $$\mu_{f}=\;\frac{T_{f}}{F_{f}\times R_{s}}$$

**Figure 3:**
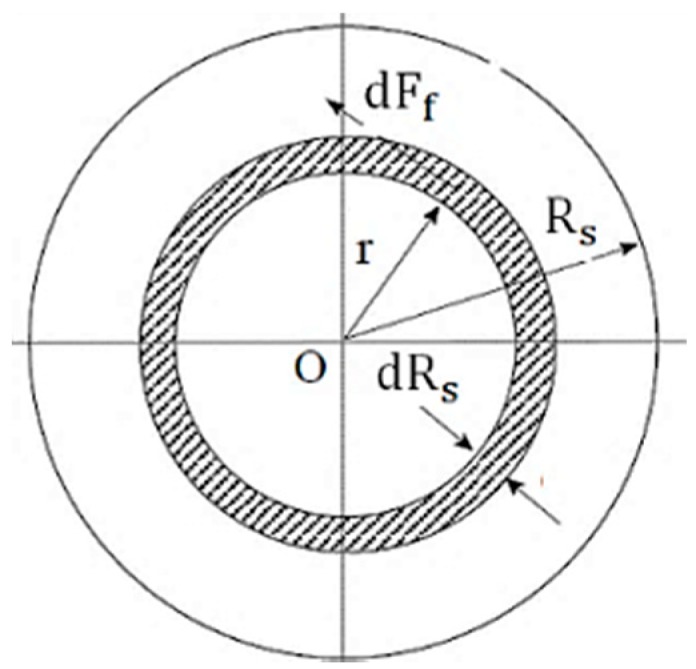
Representation of the friction force acting at the elemental radius.

The intensity of frictional heat generated during the welding of dissimilar rotating surfaces mainly depends on the friction between the two surfaces at a given point of time. The quality and strength of the welded joint are influenced by the input heat energy at the welded zone. The friction pressure and force are assumed to be the constant during the friction welding time. Figure 3 represents the friction force acting at the elemental radius, dRs. The calculated values of the friction coefficient depend on the friction force at a given spindle speed. The amount of heat generated during friction welding can be expressed as:

(2) $$H_{f}=\mathrm{Input}\;\mathrm{Heat}\;\mathrm{Energy}=\;\omega \times {\mathrm{dT}}_{f}\;\left(W\right)$$

where ${\mathrm{dT}}_{f}$ is the differential torque at radius, dr, and can be expressed as ${\mathrm{dT}}_{f}\;={\mathrm{dF}}_{f}\;\times {\;R}_{s}$ (N-m), where ${\mathrm{dF}}_{f}$ is the friction force acting at the radius, dr, and $R_{s}$ is the radius of the sample. Then, it can be defined that friction force, ${\mathrm{dF}}_{f}$, equals to the friction-coefficient multiplied by the axial-force of pressure, P, over a circle at width, dr:

(3) $${\mathrm{dF}}_{f}={\;R}_{s}\times p_{f}\times \mu_{f}\;\times 2\pi \times {\mathrm{dR}}_{s}$$

(4) $$H_{f}\;={\;R}_{s}{}^{2}\times \omega \times p_{f}\times \mu_{f}\;\times 2\pi \times {\mathrm{dF}}_{f}\times {\mathrm{dR}}_{s}$$

It can be defined that the total input heat energy at the friction welded surfaces at a distance, $R_{s}$, and thickness, ${\mathrm{dR}}_{s}$, from the axis of rotation. The total heat generated at the weld interface can be obtained by integrating the $H_{f}$ from 0 to $R_{s}$:

(5) $$\int_{0}^{R_{s}}H_{f}=\;\omega \times p_{f}\times \mu_{f}\;\times 2\pi \times {\mathrm{dF}}_{f}\;\int_{0}^{R_{s}}R_{s}{}^{2}{\;\mathrm{dR}}_{s}$$

(6) $$\int_{0}^{R_{s}}H_{f}=\;\omega \times p_{f}\times \mu_{f}\;\times 2\pi \times {\mathrm{dF}}_{f}\;\frac{R_{s}{}^{3}}{3}$$

(7) $$H_{f}=\;\omega \times p_{f}\times \mu_{f}\;\times 2\pi \times {\mathrm{dF}}_{f}\;\frac{R_{s}{}^{3}}{3}$$

(8) $$H_{f}=\frac{2\pi }{3}\;\times \omega \times p_{f}\times \mu_{f}\;\times {\mathrm{dF}}_{f}\;\frac{R_{s}{}^{3}}{3}\;\left(W\right)$$

The total friction torque by integrating with $R_{s}$ of Equation (3) is as follows:

(9) $$T_{f}=\int_{0}^{R_{s}}2\times \pi \times \mu_{f}\times {\;p}_{f}\times {\;R}_{s}{}^{2}\times {\mathrm{dR}}_{s}$$

(10) $$T_{f}=\frac{2}{3}\times \pi \times \mu_{f}\times {\;p}_{f}\times {\;R}_{s}{}^{3}\;\left(\mathrm{Nm}\right)$$

## 5. Response Surface Method-RSM

One of the remarkable optimization methods is the response surface method and it can be applied to develop the corelation model, containing effective process parameters in the friction welding process. The developed regression equation gives the response values from the dependent process parameters. In the present case, the output response was the coefficient of friction and other parameters, such as the friction pressure, friction force, friction torque, spindle speed, and friction time, were input parameters. In the first step, a suitable approximate relation between the parameters was developed by using the linear function or quadratic functions by assigning the suitable values to the parameters. The approximate first order model can be expressed as:

(11) $$Y={\;\alpha }_{0}+\alpha_{1}x_{1}+\alpha_{2}x_{2}+\alpha_{3}x_{3}+\cdots \ldots \ldots \ldots \ldots +\alpha_{k}x_{k}+\epsilon$$

The higher order equations were used for complex domains, such as curved profiles, surface curvatures, etc. These equations were capable of providing considerable approximation among all the parameters of the process. Linear approximations were more suitable to cover all effective parameters in the desired region:

(12) $$Y={\;\alpha }_{0}+\;\sum_{j=1}^{k}\alpha_{1}x_{1}+\sum_{j=1}^{k}\alpha_{2}x_{2}+\sum_{j=1}^{k}\alpha_{3}x_{3}+\cdots \ldots \ldots \ldots \ldots +\sum_{j=1}^{k}\alpha_{k}x_{k}+\epsilon$$

**Figure 4:**
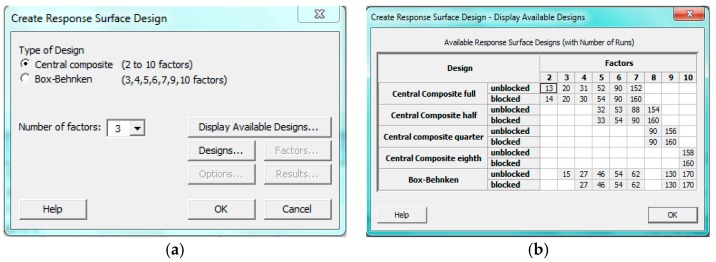
(**a**) Type of design. (**b**) Available design in the response surface design method.

**Figure 5:**
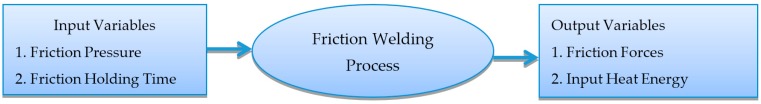
Input and output parameters of the friction welding process.

The experiments were based on the design of experiments’ techniques, i.e., composite design available in the library of statistical modelling software, MINITAB-17. Figure 4 shows the possible available design based on the levels and factors decided for optimization. The process parameters were used to develop the regression equation involving main effects and interaction of the output response with other associated process parameters. The regression equation developed was examined for the test of significance by considering all possible combinations and achieved the optimum process parameters for the friction welding. These optimum parameters were experimentally validated to get the output response. Table 4 shows the L-9 array of design of the experiment used in the friction welding experiments and the results of the response surface method performed for testing the significance at 95 % confidence level of index. The indicative terms used in this method are ‘Seq. SS’ and ‘Adj. SS’ gives the sum of squares for each term and sum of squares after deleting insignificant terms in the regression model. Similarly, ‘Adj. MS’ is the mean square achieved after deleting insignificant terms from the response equation. The ‘F’ value present in the regression equation was used to check the test hypothesis. The results indicate that the developed regression equation based on the central composite design given in Table 5 proved to be statistically adequate for the prediction of optimized parameters. It is important to note that all the terms are found to be significant on $\mu_{f}$ as the value of ‘P’ was computed to be less than 0.05. Moreover, the coefficient of the regression equation was correlated for this model and was 0.95. Critical friction welding parameters used in the experiments are presented in Table 6. It is interesting to know that all the terms significantly contributed to the response, $\mu_{f}$. The accuracy of the predicted model was determined by conducting conformity tests. In this procedure, nine test cases were completed at random by assigning coded values to the process variables, and for each combination, the output responses were determined and validated experimentally. Close fitness between the predicted and experimental values showed the adequacy of the model. Input and output parameters of the friction welding process is shown in Figure 5.

## 6. Results and Discussions

### 6.1. Effect of Friction Torque on the Coefficient of Friction

**Figure 6:**
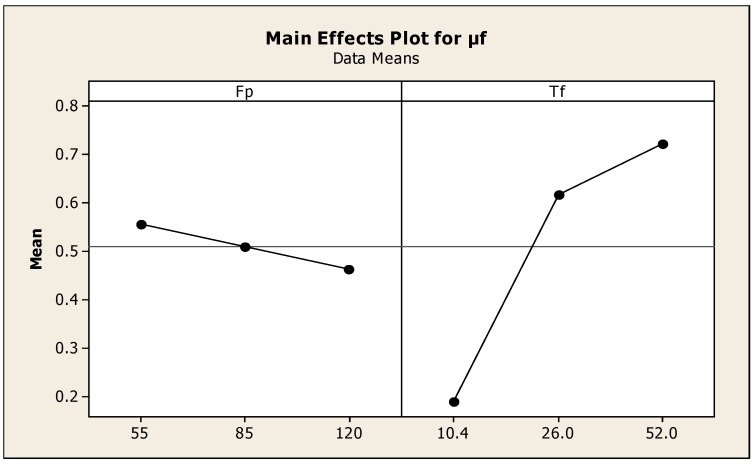
Effect of friction pressure and torque on $\mu_{f}$.

When the friction torque was increased, the measured temperatures were also increased. The plot shown in Figure 6 is the effect of friction pressure and torque on $\mu_{f}$, and reveals the optimum value of friction pressure was 85 MPa and friction torque was 25 Nm. Regression coefficients and the factors of Analysis of Variance are presented in Table 7 and Table 8. Although all measured temperatures were almost the same before the friction torque reached the initial peak, when the friction time was 0.04 s, i.e., both specimens had been rotated once, the concentric rubbing marks were observed at the half radius portion of the weld interface of both sides. When the friction time was 0.5 s, concentric overlapping marks appeared on the peripheral portions. Then, almost the whole weld interface fully developed at a friction time of 0.5 s. As the friction torque increased to the initial maximum value, the flash on the interface welded region was increased. Based on the temperatures recorded with the FLUKE IR camera results, it was observed that the heat input energy at the entire welded zone increased to the maximum value of the friction torwas performed by using experimental data to develop the relation between the friction torque and forging pressure and is expressed as, $\mu_{f}$ = 0.535 − 0.00555$F_{p}$ + 0.0155$T_{f}$. S = 0.0797562, R-Sq = 93.4% and R-Sq (adj) = 91.2%.

### 6.2. Effect of Friction Pressure on the Coefficient of Friction

**Figure 7:**
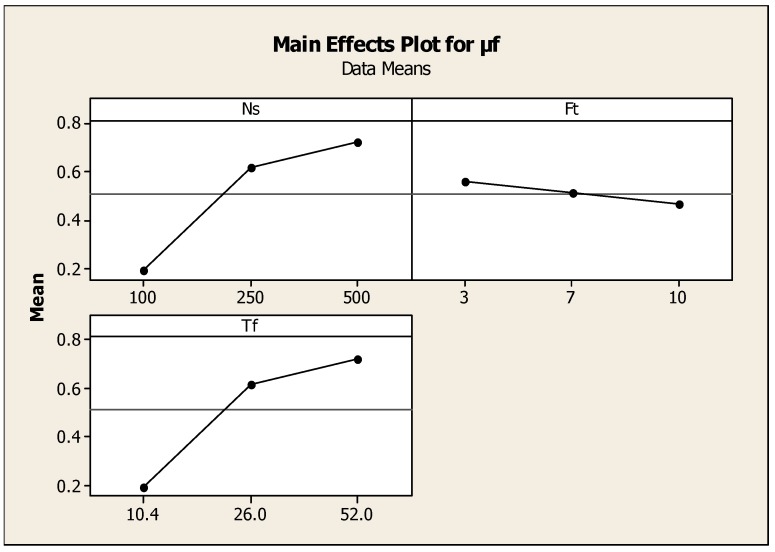
Effect of speed, torque, and friction time on $\mu_{f}$.

Copper has a narrower heat affected zone compared to aluminum when constant heat input energy was generated. This was due to the higher thermal conductivity value in copper compared to that in aluminum. In general, the friction pressure was not uniform with friction time as the two metal pieces possess different thermal conductivity. The main effect plot shown in Figure 7 is the effect of speed, torque, and friction time on $\mu_{f}$ and reveals that the optimum value of friction torques was 24 Nm and friction time was 7 s with a spindle speed of 240 rpm. Table 9 and Table 10 show the regression coefficients and Anova results. The area of welded interface between the two metals changed during welding and led to the variation of the axial pressure. Therefore, when the friction pressure changed during friction welding due to the variation of the contact area, and leading to different input frictional heat input, this gave rise to the initiation of cracks and fractures of the surface. The coefficient of friction varied widely with heat input energy and friction pressure. The increase in the friction pressure increased temperature on the interface surfaces and the coefficient of friction was considerably reduced. This trend was observed for the value of coefficient, $\mu_{f}$ = 0.12, $F_{t}$ = 10 s, and $F_{p}$ = 120 MPa. The regression equation was expressed as $\mu_{f}$ = 0.488 − 0.000032$F_{f}$ + 0.00161$N_{s}$ − 0.0181$F_{t}$. Where S = 0.0869256, R-Sq = 93.5%, R-Sq (adj) = 89.5%.

### 6.3. Effect of Friction Time on the Coefficient of Friction

**Figure 8:**
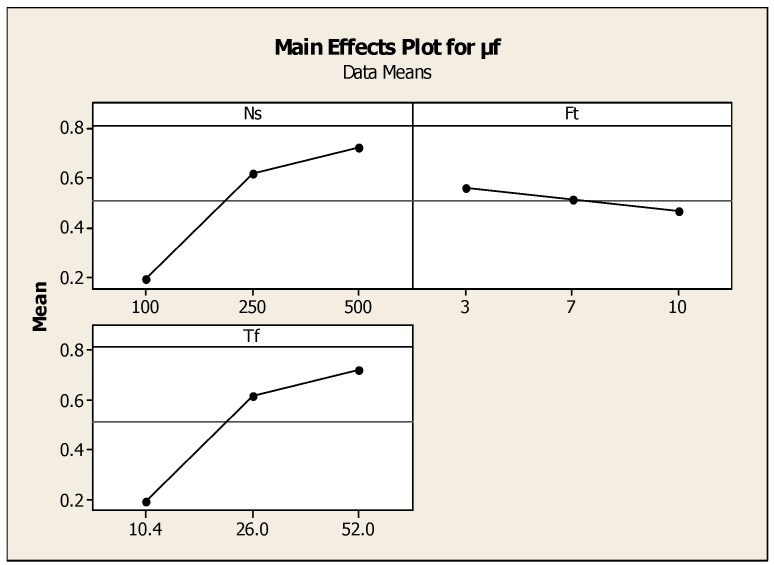
Effect of torque and friction time on $\mu_{f}$.

From the main effects plot shown in Figure 8, the effect of torque and friction time on $\mu_{f}$ are shown. When friction time was increased, the fractured surfaces decreased from high peaks to normal interface surface, which means the uniform welding at the interface was achieved. The optimized friction time was estimated to be in the range of 3 s–7 s, under which the good quality of the welded joint was possible. In case of less friction time, the crack growth initiates during the initial period and extended to periphery. Ruptured patterns were observed on the fracture surfaces of the welded interface at the friction time less than 3 s and observed river patterns were confirmed. Table 11 and Table 12 show the regression coefficients and Anova results. There were crack detections and porosities distributed on the interfacial fractured surface. It can be concluded that less friction welding time leads to increased fractured surfaces when compared to the extended friction time conditions. The regression equation was $\mu_{f}$ = 0.488 − 0.000032$F_{f}$ + 0.00161$N_{s}$ − 0.0181$F_{t}$.

### 6.4. Investigation of Heat Flux Generated and Spindle Speed on the Coefficient of Friction

**Figure 9:**
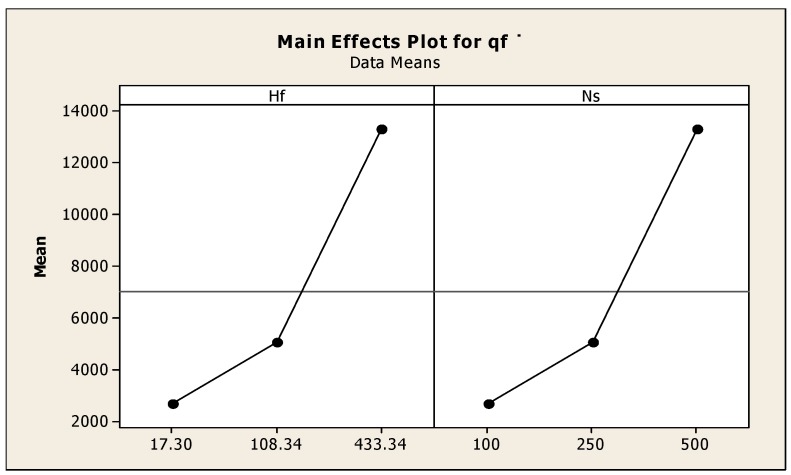
Effect of input heat energy and speed on $\mu_{f}$.

From the main effects plot shown in Figure 9, the effect of the input heat energy and speed on $\mu_{f}$ are observed. When the friction time increased, thereby heat input energy increased and results in good weld interface joint. The optimized input heat energy was 120 W with a spindle speed nearer to 350 rpm, and under these combinations, good quality of the welded joint was possible. In case of less friction time, the crack growth initiated during the initial period and extended to periphery. River patterns were observed on the fracture surfaces of the welded interface at the friction time less than 3 s and observed river patterns were confirmed. There were crack detections and porosities distributed on the interfacial fractured surface. Table 13 and Table 14 show the regression coefficients obtained and Anova results. It can be concluded that less friction welding time leads to the relatively more fractured surfaces when compared to the extended friction time conditions. The regression equation was $\mu_{f}$ = −0.206 − 0.000018$q_{f}$ −0.00304$H_{f}$ + 0.00498$N_{s}$, S = 0.0849933, R-Sq = 93.7%, R-Sq (adj) = 90.0%.

### 6.5. Investigation of Heat Flux Generated, Friction Pressure, and Friction Torque on the Coefficient of Friction

**Figure 10:**
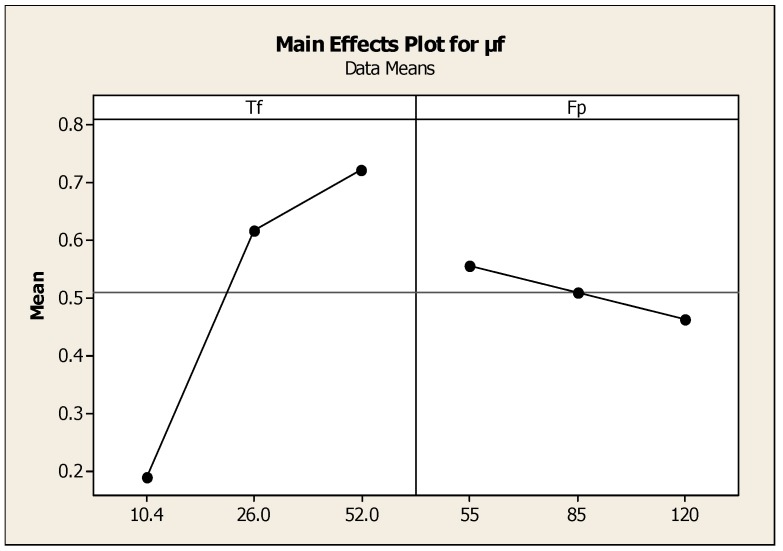
Effect of friction pressure and torque on $\mu_{f}$.

**Figure 11:**
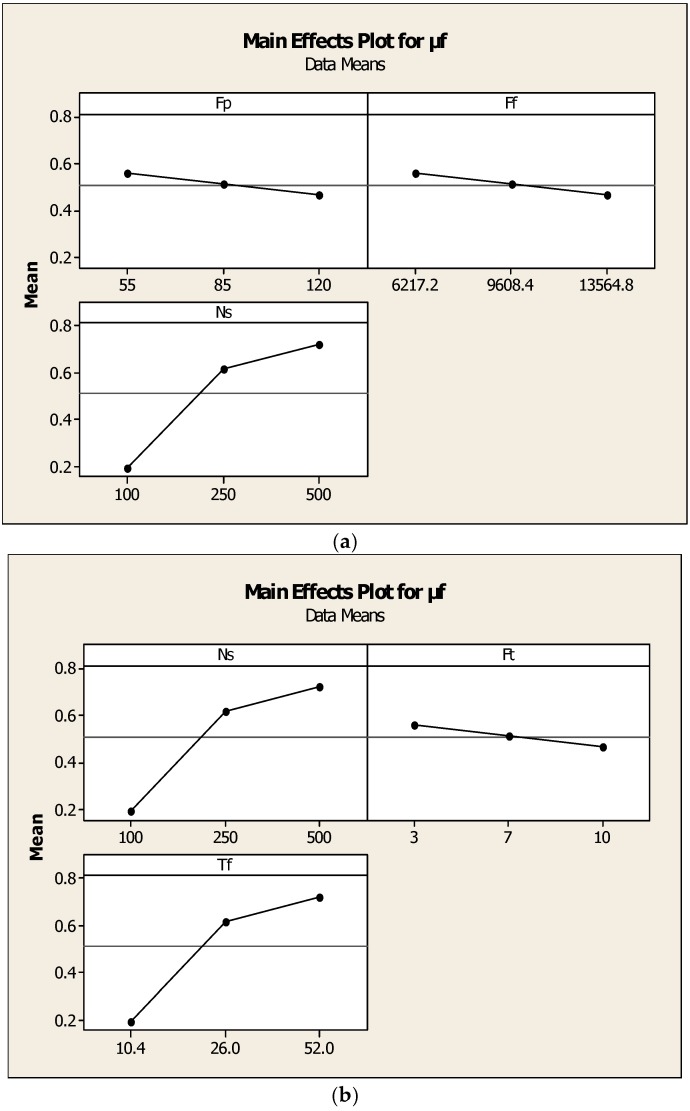
(**a**) Effect of speed, friction pressure. (**b**) Effect of speed, friction torque, and friction force on $\mu_{f}$.

The main effects plot shown in Figure 10 reveals the effect of friction pressure and torque on $\mu_{f}$, and as the friction torque was increased, the fractured surfaces decreased even on the periphery and at the center of the interface surface. If the friction torque along with the friction pressure increased, consequently, the welded surface had uniform metal bonding. The optimized friction torque was found to be 25 Nm and the friction pressure was 85 N/mm^2^. These optimum parameters were achieved at the 95% confidence limit. In Figure 11a, the main effect of speed, friction pressure, and Figure 11b the effect of speed, friction torque, and friction force on $\mu_{f}$ are shown. Due to more heat input energy, the fractured patterns had lesser intensity on the fracture surfaces of the welded interface at the friction time less than 3 s and observed river patterns were confirmed. There were crack detections and porosities distributed on the interfacial fractured surface. Table 15 and Table 16 show the regression coefficients obtained and Anova results. It can be concluded that less friction welding time leads to relatively more fractured surfaces when compared to the extended friction time conditions The regression equation was $\mu_{f}$ = 0.478 − 0.000007$q_{f}$ − 0.00477$F_{p}$ + 0.0168$T_{f}$, S = 0.0799830, R-Sq = 94.5%, R-Sq (adj) = 91.1%

**Figure 12:**
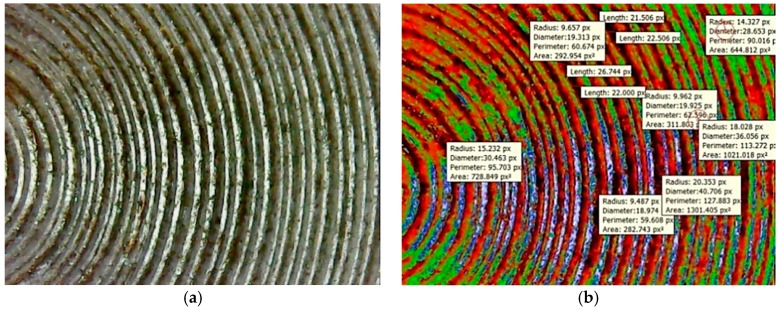
(**a**) Friction welded tracks at the welded interface. (**b**) Segment based analysis image.

At the weld interface, fracture surfaces are shown in Figure 12a. It can be clearly seen that the major cracks and fractured surfaces at the periphery were not involved in the welding. At the center portion of the weld interface, there was more metal penetration in the bonding and the fractured surface varied from that of smoother ones to cracks regions. The river pattern shows that the weld interface was formed through rotational friction under the influence of more friction pressure.

## 7. Macro Examination of Aluminum-Copper Welded Interface by Using IST

**Figure 13:**
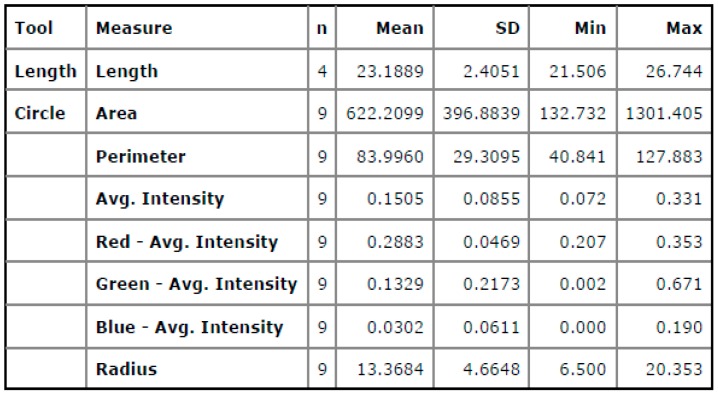
Screen shot of the statistical measurements of the segments at the optimized parameters.

The propagation of crack growth and fractured surfaces at the welded interface are crucial to identify. It is difficult to measure the crack length of the open fractured surface based on the available images with dissimilar metals and surface topologies using Non Destructive Testing (NDT), thermography, etc. In such cases, it is more feasible to identify crucial crack lengths and fractured surfaces by means of the image segmentation technique. Based on the friction welding experiments conducted, some of the surface flaws were detected in terms of the crack length and fractured surface area, and they are expressed in different colored intensities, such as red, green, and blue, for quantification of surface defects in pixels. Based on the analysis, it is concluded that in Figure 12a, the contrasting color between the welded region and cracks is high. In Figure 13, the screen shot of colored regions obtained from the DIGIMIZER software gives different colored intensities, which were given based on statistical measurements of the segments at the crack lengths. The background surface includes a lot of dark and colored spots, which affects image segmentation intensities. The crack length was extracted in terms of intensities, as shown in Figure 12b, that had a high intensity of fractured surface area (1301.405 Px^2^) as compared to the background crack area (282.743 Px^2^), with a less average blue colored mean intensity of 0.0302.

**Figure 14:**
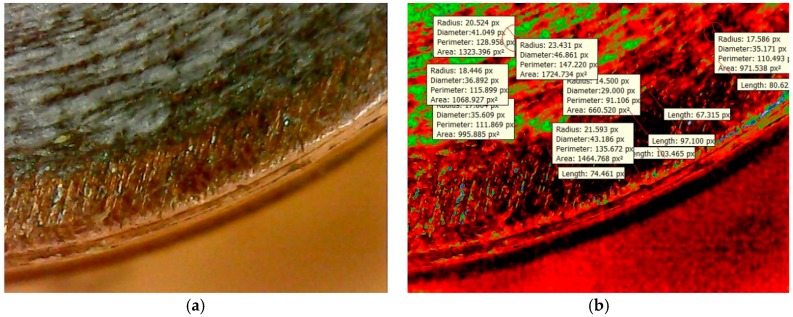
(**a**) Al-Cu fractured surface of the welded interface. (**b**) Segment based analysis of the fracture surface.

**Figure 15:**
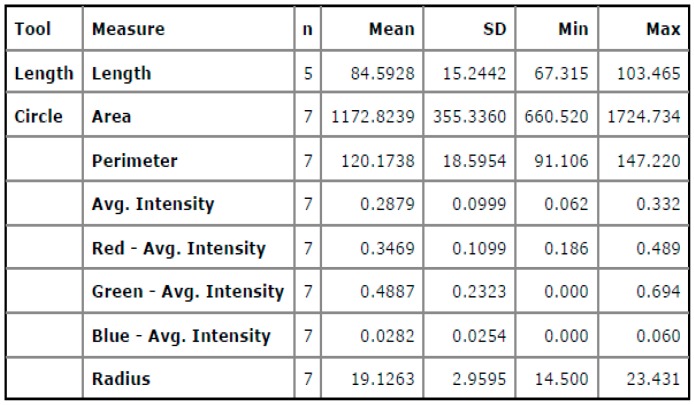
Screen shot of the statistical measurements of the segments at the fractured surface.

At the close vicinity of edges, large numbers of cracks are identified when compared to the center region of the welded interface with high colored intensities. In Figure 14b, the intensity of green color is found to be more when compared to red colored intensity, which means near edges’ input heat energy was less due to the greater crack lengths observed. In Figure 15, different colored intensities are given based on statistical measurements of the segments at the fractured surface. The average blue colored intensities of 0.028 is less, which is a measure of the good quality of the weld. The maximum crack length was found to be 103.465 Px. The maximum fractured surface area was found to be 1724.734 Px^2^ as shown in Figure 14b. In Figure 15, the screen shot of colored regions obtained from the DIGIMIZER software gives different colored intensities at the fractured surface based on statistical measurements.

**Figure 16:**
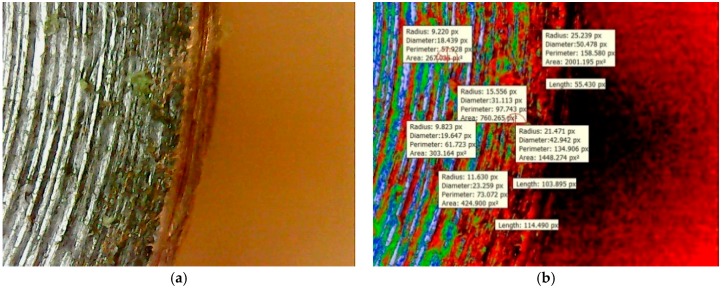
(**a**) Al-Cu welded interface at the edge. (**b**) Segment based analysis of the crack length.

**Figure 17:**
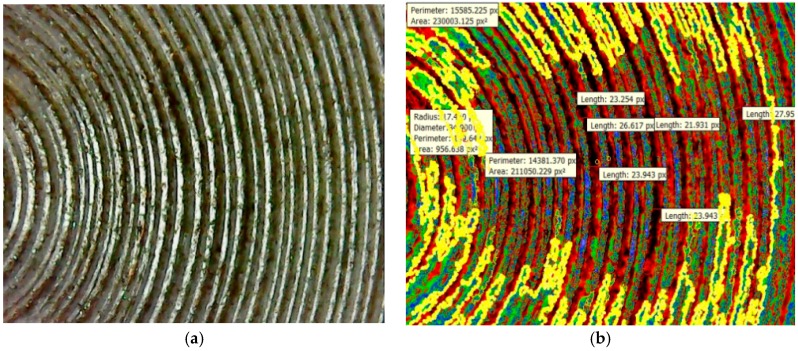
(**a**) Peaks and valleys at Al-Cu interface. (**b**) Segment based analysis of the welded region.

It was found on the outer periphery that there are many pores in close vicinity of the metallic interface due to uneven friction forces acting on the welded surfaces at high rotational speeds that leads to porosities. The mean red colored intensities of 0.4603 was more when compared to other welded regions. The maximum crack length was found to be 103.895 Px as shown in Figure 16b. The mean green colored intensity was found to be less when compared to the red colored intensity. In Table 14, different mean colored intensities are given based on statistical measurements of the segments as porosities. Figure 17 depicts the information about the different intensities at the welded zones with the help of statistical measurements of the segments at the fractured surface. The experimental validation was carried out for the optimum results of the process parameters and confirmed the maximum welded region (yellow color) having an area of 230,003.125 Px^2^. The mean welded area was found to be 212,357.46 Px^2^. Different colored intensities are given for friction welded tracks at the interface having mean crack lengths of 24.60 Px based on statistical measurements of the segments at the welded interface.

The maximum crack length was found to be 23.254 Px, which was less when compared to other parameters of the friction welding process. The screen shot shows the different regions of the flaws at the interface in terms of statistical measurements of the segments’ intensity. The maximum crack length was found to be 23.25 Px for the black colored intensity region, which was a lesser percentage when compared to other blue colored intensities. This black colored region is the subsurface of the interface and contains less number of micro cracks, and in this region, the maximum crack length was found to be 23.25 Px, as shown in Figure 17b. In the blue colored region, the maximum crack length was found to be 26.61 Px, and this region is the mid surface region at the welded interface. The ellow colored intensity region is considered to be the peak zone where the surface cracks were found to be more. In this yellow colored region, the maximum crack length was found to be 27.95 Px.

## 8. Conclusions

In the present research, the factors influencing the friction welding process were studied based on the response surface method. The factors considered were the spindle speed, friction pressure, friction force, torque, and friction time. Friction time played a vital role for each experiment and demonstrated that as the friction time increased, more heat input energy overcame the friction and penetrated more into the weld interface without surface cracks. A regression equation correlation was then proposed to evaluate the optimal friction time for a wide range of friction welding parameters. Once the optimal operating conditions were obtained for friction pressure by the response surface method, experimental tests were carried out. The weld quality tests were conclusive and demonstrate that the appearance of the fractured surface occurred at the interface with more peaks and porosities for less friction pressure values. The effect of the experimental tests and results of optimization were in good agreement for the crack length and fractured surface for the lesser values of the coefficient of friction due to more friction forces acting at the interfaces. Experimental validation was carried out by selecting the optimized friction welding parameters and comparing the results obtained from IST micrograph for a good welded joint quality. The morphology of river patterns appeared on fractured surfaces for the lesser friction pressure values and were confirmed with the micrograph obtained from segmentation analysis having more pixels in the fractured surfaces. The appearance of micro cracks at the interface were due to thermo-mechanical coupling effects with less spindle speed and more friction forces during friction welding. The segmentation analysis technique proposed in this work is useful for better controlling the friction welding method. The interface zone, where the fractured surface and crack length affects the strength of the welded joint, are more dependent on the friction pressure, heat input energy, and heat flux generated during friction welding. Therefore, the optimized parameters and regression analysis proposed the effective parameters for a good welded joint. The predicted optimized parameters of the friction welding were in good agreement with the experimental validations. Further studies on crack detection and study of the interfacial fractured surface can be done on additives of reinforcement powder, such as TiO_2_ and SiC, and the effect of these additions on the coefficient of friction with respect to different process parameters.

## Figures and Tables

**Table 1:** Chemical composition of Al-Cu dissimilar metals used in the FW process (weight %).

Metal	Si	Cu	Fe	Mn	Ni	Mg	Sn	Zn	Pb	Al
Al	0.85	0.35	0.2	0.05	0.05	0.03	0.03	0.3	-	Remaining content
Cu	-	Balance	0.04	0.05	0.05	1.83	0.03	0.06	0.04	-

**Table 2:** Mechanical properties of metals used in the friction welding experiment.

Metal	Density, g/cm^3^	Tensile Strength, MPa	Young’s Modulus, GPa	Brinell Hardness
Cu	8.9	220	104	75
Al	2.7	170	78	48

**Table 3:** Friction welding factors and their design levels.

S. No	Factors	Symbol	Units	Levels		
-				−1	0	1
				Low	Middle	High
1	Friction Force	$F_{f}$	N	6217.2	9608.4	13,564.8
2	Speed	$N_{s}$	RPM	100	500	1000
3	Friction Pressure	$F_{p}$	MPa	55	85	120
4	Friction Time	$F_{t}$	s	3	7	10

**Table 4:** L-9 Array for the design of the experiments.

Experiment. No	Actual Values			Coded Values			Output Response	
	$N_{s}$	$F_{p}$	$F_{t}$	$N_{s}$	$F_{p}$	$F_{t}$	$\mu_{f}$	$\dot{q_{f}}$ $,\;W/mm^{2}$
1	100	55	3	−1	−1	−1	0.27	1727
2	250	85	7	1	−1	−1	0.45	6672
3	500	120	10	−1	1	−1	0.63	18,840
4	250	55	3	1	1	−1	0.70	4317.5
5	500	85	7	−1	−1	−1	0.90	2239.8
6	100	120	10	1	−1	−1	0.12	3768
7	250	55	3	−1	1	−1	0.70	4317.5
8	100	85	7	1	1	−1	0.18	2669
9	500	120	10	−1	−1	1	0.64	18,840

**Table 5:** Central composite design.

Factors	3
Base runs	20
Base blocks	1
Two-level factorial	Full factorial
Cube points	8
Center points in cube	6
Axial points	6
Center points in axial	0
Alpha	1.68179
Replicates	1
Total runs	20
Total blocks	1

**Table 6:** Critical friction welding parameters used in the experiments.

Runs	$F_{p},\;N/{\mathrm{mm}}^{2}$	$F_{f},\;N$	$T_{f},\;\mathrm{Nm}$	$F_{t},\;s$	$N_{s},\;RPM$	$H_{f},W$	$\mu_{f}$	$\dot{q_{f}}$ $,\;W/mm^{2}$
1	55	6217.2	10.4	3	100	17.3	0.27	1727
2	85	9608.4	26	7	250	108.34	0.45	6672
3	120	13,564.8	52	10	500	433.34	0.63	18,840
4	55	6217.2	26	3	250	108.34	0.70	4317.5
5	85	9608.4	52	7	500	433.34	0.90	2239.8
6	120	13,564.8	10.4	10	100	17.3	0.12	3768
7	55	6217.2	26	3	250	108.34	0.70	4317.5
8	85	9608.4	10.4	7	100	17.3	0.18	2669
9	120	13,564.8	52	10	500	433.34	0.64	18,840

**Table 7:** Regression equation coefficients.

Predictor	Coef	SE Coef	T	P
Constant	0.53517	0.09138	5.86	0.001
$F_{p}$	−0.00555	0.001098	−5.052	0.002
$T_{f}$	0.015471	0.001700	9.10	0.000

**Table 8:** Analysis of variance.

Source	DF	SS	MS	F	P
Regression	2	0.5396	0.26982	42.42	0.000
Residual Error	6	0.0381	0.00636	-	-
Total	8	0.5778	-	-	-

**Table 9:** Regression equation coefficients.

Predictor	Coef	SE Coef	T	P
Constant	0.4878	0.2319	2.10	0.089
$\dot{q_{f}}$	−0.000031	0.00007	−0.42	0.693
$H_{f}$	0.00160	0.00019	8.35	0.000
$N_{s}$	−0.0180	0.0800	−0.23	0.830

**Table 10:** Analysis of variance.

Source	DF	SS	MS	F	P
Regression	3	0.540	0.180	23.82	0.002
Residual Error	5	0.037	0.007	-	-
Total	8	0.577	-	-	-

**Table 11:** Regression equation coefficients.

Predictor	Coef	SE Coef	T	P
Constant	0.4878	0.2319	2.10	0.089
$\dot{q_{f}}$	−0.00003199	0.000076	−0.4	0.69
$H_{f}$	0.0016098	0.00019	8.35	0.000
$N_{s}$	−0.01808	0.080	−0.2	0.83

**Table 12:** Analysis of variance.

Source	DF	SS	MS	F	P
Regression	3	0.5400	0.18001	23.82	0.002
Residual Error	5	0.0377	0.00756	-	-
Total	8	0.5778	-	-	-

**Table 13:** Regression equation coefficients.

Predictor	Coef	SE Coef	T	P
Constant	−0.2055	0.1228	−1.67	0.155
$q_{f}$	−0.000018	0.0000061	−2.97	0.031
$H_{f}$	−0.003039	0.0009467	−3.21	0.024
$N_{s}$	0.004981	0.001012	4.92	0.004

**Table 14:** Analysis of variance.

Source	DF	SS	MS	F	P
Regression	3	0.5416	0.1805	25.00	0.002
Residual Error	5	0.03612	0.00722	-	-
Total	8	0.57780	-	-	-

**Table 15:** Regression equation coefficients.

Predictor	Coef	SE Coef	T	P
Constant	0.4775	0.1088	4.39	0.007
$q_{f}$	−0.00000696	0.00000708	−0.98	0.371
$H_{f}$	−0.004767	0.001360	−3.51	0.017
$N_{s}$	0.016785	0.002167	7.75	0.001

**Table 16:** Analysis of variance.

Source	DF	SS	MS	F	P
Regression	3	0.54581	0.18194	28.44	0.001
Residual Error	5	0.03199	0.00640	-	-
Total	8	0.57780	-	-	-

## Nomenclature

$T_{f}$	Friction Torque (Nm)
$R_{s}$	Radius of the weld metal,mm
$F_{f}$	Friction Force (N)
$N_{s}$	Spindle Speed,RPM
$\mu_{f}$	Coefficient of Friction
$F_{p}$	$Friction\;Pressure,\;N/mm^{2}$
$F_{t}$	Friction Time, seconds
R-Sq	Squared Residue
R-Sq (adj)	Adjoint Squared Residue
$H_{f}$	Input Heat Energy (W)
$\dot{q_{f}}$	$Heat\;Flux\;generated\;due\;to\;friction\;\left(W/m^{2}\right)$
DF	Degree of Freedom
SS	Sum of Squares Value
MS	Mean Square Value
F	F-Value
P	P-Value
